# Elevated cholesteryl ester transfer and phospholipid transfer proteins aggravated psoriasis in imiquimod-induced mouse models

**DOI:** 10.1186/s12944-022-01684-0

**Published:** 2022-08-18

**Authors:** Jun Chen, Haihua Qi, Lijun Liu, Yandong Niu, Shuping Yu, Shucun Qin, Lei He

**Affiliations:** 1 Department of Geriatrics, The Affiliated Hospital of Chengde Medical University Chengde, 067000 Hebei, China; 2 Department of Dermatology and Venerology, The Affiliated Hospital of Chengde Medical University, Chengde, 067000 Hebei China; 3 Institute of Atherosclerosis, Shandong First Medical University, Tai’an, 271000 Shandong China

**Keywords:** High-density lipoprotein, Psoriasis, Phospholipid transfer protein, Cholesteryl ester transfer protein

## Abstract

**Background:**

Psoriasis is a chronic inflammatory skin disorder related to dyslipidemia, with decreased high-density lipoprotein (HDL). Various cell types express phospholipid transfer protein (PLTP) as well as cholesteryl ester transfer protein (CETP). Their elevated levels among transgenic (Tg) mice led to reduced HDL and a higher risk of atherosclerosis (AS). This study examined whether elevated CETP and PLTP could aggravate psoriasis in a psoriasis vulgaris mouse model.

**Methods:**

The back skins of CETP-Tg, PLTP-Tg, and C57BL/6 male mice, aged six to 8 weeks, were shaved for imiquimod cream (IMQ) (5%) treatment for five consecutive days. The clinical pathological parameters were rated independently using the modified target lesion psoriasis severity score. The skin sections stained with hematoxylin-eosin were scored by the Baker score. Epidermal thickening and differentiation and inflammatory factor infiltration were determined by immunohistochemistry. Inflammatory cytokine levels were measured using quantitative reverse transcription-polymerase chain reaction (RT–PCR) and enzyme-linked immunosorbent assay (ELISA) kits. This work employed SPSS Statistics Version to conduct statistical analyses.

**Results:**

In this study, CETP-Tg and PLTP-Tg mice had higher clinical and histological scores than wild-type (WT) mice. Immunohistochemistry of the epidermis and dermis revealed a high proportion of proliferating cell nuclear antigen (PCNA) positivity within psoriatic skin lesions of CETP-Tg and PLTP-Tg mice compared with WT mice. Interferon-α (IFN-α), interleukin-1β (IL-1β), IL-6, IL-17A, IL-17F, IL-22, and IL-23p19 mRNA levels increased within CETP-Tg and PLTP-Tg mice compared with WT counterparts. In comparison with WT mice, plasma tumor necrosis factor-α (TNF-α) levels, rather than IL-6 levels, were increased in CETP-Tg and PLTP-Tg mice.

**Conclusions:**

Elevated CETP and PLTP aggravate psoriasis in a imiquimod-induced mouse model.

## Background

Psoriasis is a chronic and recurrent inflammatory immune disease that has a certain effect on skin and additional tissues, including the cardiovascular system [1, 2]. Conventional risk factors for cardiovascular diseases (CVDs), including dyslipidemia, are frequently detected in psoriasis patients [3, 4]. Psoriasis patients with dyslipidemia have decreased high-density lipoprotein cholesterol (HDL-C) content [5]. According to an increasing number of studies, psoriasis changes cholesterol efflux ability and HDL composition, while these are restored through treatment against psoriasis [6, 7]. Furthermore, a previous study revealed that psoriasis reduced HDL’s anti-inflammatory and antioxidative characteristics.

Plasma lipid transfer proteins, such as phospholipid transfer protein (PLTP) and cholesteryl ester transfer protein (CETP), are related to HDL metabolism. CETP, the plasma glycoprotein, is generated within some tissue types [8]. CETP transfers cholesteryl ester into lipoproteins containing apolipoprotein B (apo B) from mature spherical HDL [9, 10]. CETP reduces HDL cholesterol levels. In several human [11, 12] and animal studies [13, 14], CETP facilitated the development of atherosclerosis and is a major inhibited target for reducing atherosclerosis risk [15]. Plasma CETP expression cannot be detected within mice [16]. After introducing the human CETP transgene in mice, plasma HDL content declined, which is related to accelerated HDL cholesteryl ester (CE) catabolism because of the promotion of liver cancer [17, 18]. PLTP accounts for a lipid transfer/lipopolysaccharide-binding protein family member, such as CETP [19]. It is a monomeric protein [20] that is distributed in some cells. It is related to HDL in plasma. Higher PLTP expression within transgenic (Tg) mice accelerates atherosclerosis (AS) and decreases HDL [21].

Psoriatic HDL decreases and gradually loses normal biological activities. Increased CETP and PLTP levels induce AS development and HDL reduction. Therefore, whether CETP and PLTP affect psoriasis was observed in imiquimod-induced mouse models.

## Methods

### Mice

Thirty male mice aged six to 8 weeks were recruited. Dr. Xiancheng Jiang provided the CETP-Tg and PLTP-Tg C57BL/6 mice. This work utilized PLTP and CETP heterozygous mouse offspring littermates as CETP-Tg, PLTP-Tg, and WT mice, with six animals in each group. All animals were raised with a chow diet (KEAOXIELI FEED. Co. LTD, Beijing, China) with free access to water. They were raised within a controllable temperature and humidity under a 12-h/12-h light-dark cycle. This work acquired 6- to 8-week-old C57BL/6 male mice from Peking University’s Experimental Animal Center in Beijing, China. All experiments were approved by the Laboratory Animal Care Committee of Shandong First Medical University. Each animal experimental procedure was carried out following the Guides of Care and Use of Laboratory Animals of Shandong First Medical University (Ethical approval No SYXK20190022).

### Imiquimod-induced psoriasis model and assessment

The present experiment was carried out following the imiquimod-mediated psoriatic lesion mouse model [22, 23]. The back skin in each animal was shaved and consecutively exposed to 5-day treatment of imiquimod cream (IMQ) (5%) at 62.5 mg (Aldara; 3 M Pharmaceuticals, St Paul, USA). Disease clinical parameters were rated independently for the animals using the modified target lesion psoriasis severity score (TLPSS), which included a cumulative score for scaling, erythema, and thickness at the 0–4 scale, where four represented the most serious level. The sum of these three separate assessments was made, yielding a total score of 0–12. Overall, 0 represents the absence of alteration, while 1–4 indicate 0–25%, 25–50%, 50–75%, and 75–100% of affected shaved area, respectively [24].

### Tissues and samples

Twenty-four hours after the last imiquimod treatment and with no 12-h dietary exposure, blood was collected in the mouse retro-orbital sinus. Then, the plasma was used for the experiment or stored at − 80 °C. Later, each animal was killed through cervical dislocation, while some sections of dorsal skin were extracted, followed by fixation with 4% paraformaldehyde, processing, paraffin embedding, slicing to 5-μm sections, and hematoxylin-eosin (HE) staining. Additionally, part of the dorsal skin was subjected to snap-freezing within liquid nitrogen, followed by preservation at − 80 °C prior to use.

### Histological scoring

Skin sections stained with HE were evaluated by two blinded dermatopathologists. As shown in Table 1, all samples were scored on the Baker score criteria for microabscess, rete ridge lengthening, parakeratosis, hyperkeratosis, thinning above papillae, acanthosis, lack of a granular layer, papillary papillae congestion, and lymphocytic infiltrate [25].

**Table 1:** Criteria of histopathological scores

	Pathological alteration	Score
Corneous layer	Munro abscess	1.5
	Hyperkeratosis	0.5
	Parakeratosis	1.0
Epidermis	Lengthening of rete ridges	0.5–1.5
	Lack of granular layer	1.0
	Acantosis	1.0
Dermis	Lymphocytic infiltrate	0.5–1.5
	Papillary papillae congestion	1.0
	Thinning above papillae	0.5

### Immunohistochemistry analysis

Paraffin sections (5 μm) were stained using certain antibodies against proliferating cell nuclear antigen (PCNA) (Santa Cruz Biotechnology, CA, USA). Briefly, each paraffin section was subjected to xylene deparaffinization twice, followed by gradient ethanol rehydration. Thereafter, each section was subjected to a 5-min incubation using PBS-diluted 0.5% hydrogen peroxide to inhibit endogenous peroxidase activity. Epitope retrieval under heat induction was conducted within 10 mM citrate buffer for 15 min at 95 °C. Later, sections were blocked using 1.5% blocking serum, followed by 1-h antibody incubation under ambient temperature and later biotinylated horseradish peroxidase-conjugated secondary antibody incubation. The reaction was developed with a 3,3-diaminobenzidine chromogen solution. Each section was later subjected to counterstaining using hematoxylin and then mounted and subjected to histological analysis. Sections subjected to incubation without primary antibody were used as negative controls (NCs). A microscope (Olympus, Tokyo, Japan) was utilized to capture images. A reviewer blinded to the experiment was responsible for counting the positive cell number, and the mean was calculated [22].

### Real-time quantitative PCR (qRT–PCR)

Following a quality check with spectroscopy, this work separated total cellular RNA in back skin using TRIzol (Invitrogen, Grand Island, NY, USA). Later, a reverse transcriptase reaction to convert isolated mRNA to cDNA was conducted using a QuantScript RT kit (TianGen Biotech, Beijing, China) according to the manufacturer’s instructions. In addition, in this study, the SYBR-green PCR master mix kit (TianGen Biotech, Beijing, China) was utilized for qRT–PCR. GAPDH was used as a normalization control. Table 2 lists all primers utilized in qRT–PCR. In addition, Rotor-gene Q software version 1.7 (Qiagen, Valencia, CA, USA) was employed for data analysis. The 2^-DDCt^ method was applied to determine mRNA expression.

**Table 2:** Primers utilized in qRT–PCR

Gene	PCR primer
IL-17A	F: TATCCCTCTGTGATCTGGGAAG R: ATCTTCTCGACCCTGAAAGTGA
IL-17F	F: CAAGAAATCCTGGTCCTTCG R: GAGCATCTTCTCCAACCTGAA
IL-22	F: TCATTCAAAGGTGGCCTCAG R: CAAGGGGAAGGAGAGCCTTA
IL-23	F: CACCTCCCTACTAGGACTCAGC R: CTGCCACTGCTGACTAGAAC
IL-1	F: ACTGTTTCTAATGCCTTCCC R: ATGGTTTCTTGTGACCCTGA
IL-6	F: ACCACGGCCTTCCCTACTTC R: CTCATTTCCACGATTTCCCAG
INF-α	F: AGTGAGCTGACCCAGCAGAT R: CAGGGGCTGTGTTTCTTCTC
GAPDH	F: TGACGTGCCGCCTGGAGAAA R: AGTGTAGCCCAAGATGCCCTTCAG

### Plasma cytokine determination

This work measured plasma IL-6, IL-17A, and TNF-α levels using ELISA kits (Blue gene, Shanghai, China) in line with specific protocols.

### Statistical analysis

Data are displayed as the means±SDs. This work applied Student’s t test relative to both groups, where the nonparametric Mann–Whitney U test (two-tailed) was utilized in abnormally distributed data. *P* < 0.05 stood for significance level. It was employed with SPSS 22.0 to conduct statistical analyses.

## Results

### Clinical assessment of back skin

**Fig. 1:**
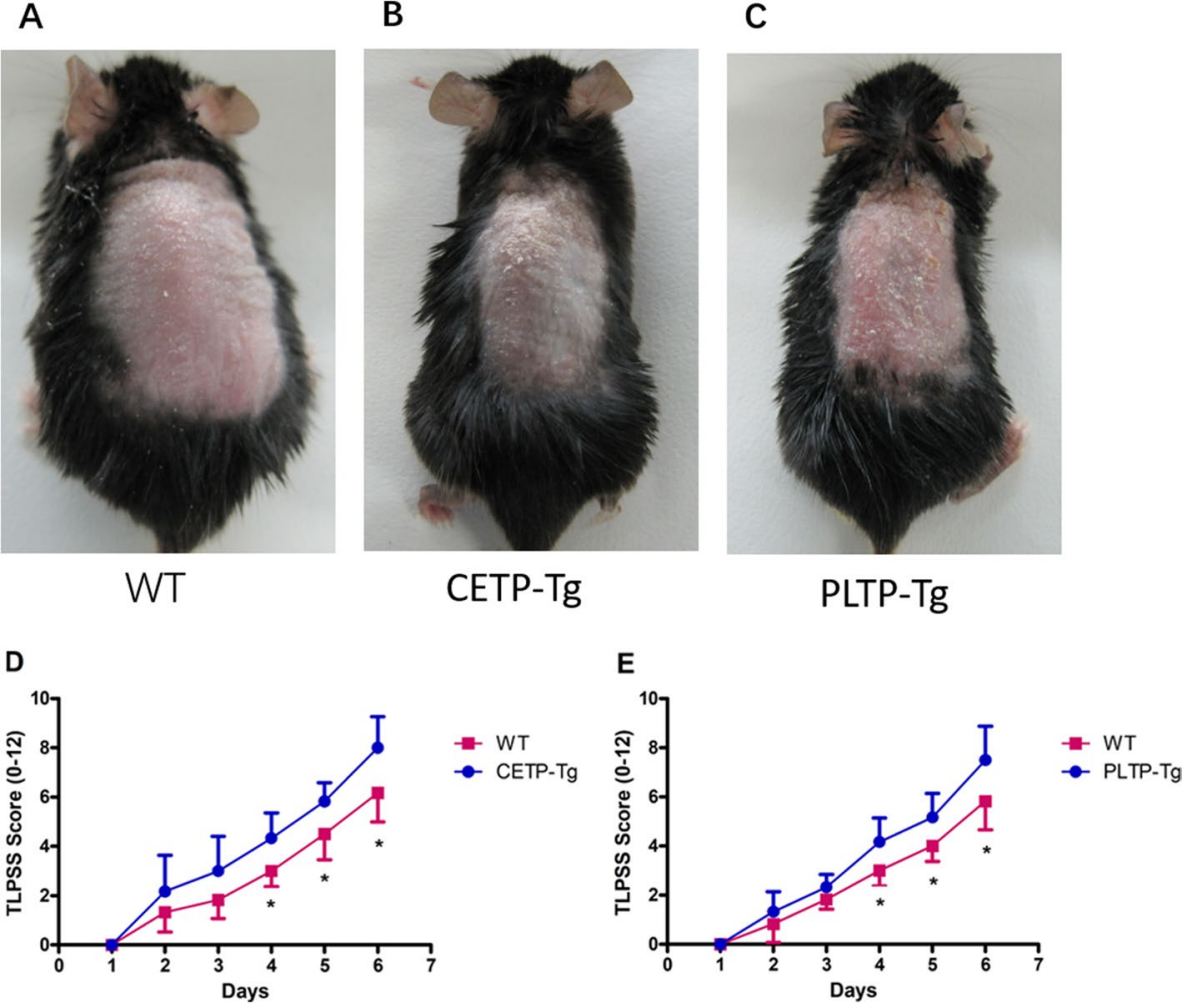
Imiquimod-mediated skin inflammation in a psoriasis mouse model. Back skin shaved in CETP-Tg, PLTP-Tg, and WT mice was exposed to IMQ cream every day. **A**–**C** Phenotypes of the back skin of mice following treatment for five days consecutively. **D**–**E** Back skin clinical cumulative score (TLPSS; erythema + scaling + thickness). Symbols represent the mean ± SD; *n* = 6/group; *P < 0.05 vs. WT group; IMQ, imquimod; CETP, cholesteryl ester transfer protein; PLTP, phospholipid transfer protein; TLPSS, target lesion psoriasis severity score; WT, wild-type; Tg, transgenic

Five consecutive days following the initiation of IMQ use, CETP-Tg and PLTP-Tg mice’s back skin displayed more signs of erythema, thickening, and scaling than WT mice. Figure 1A-C depicts typical examples. TLPSS was higher in CETP-Tg and PLTP-Tg mice than in WT mice (Fig. 1D, E).

### Back skin histology and immunohistochemistry

**Fig. 2:**
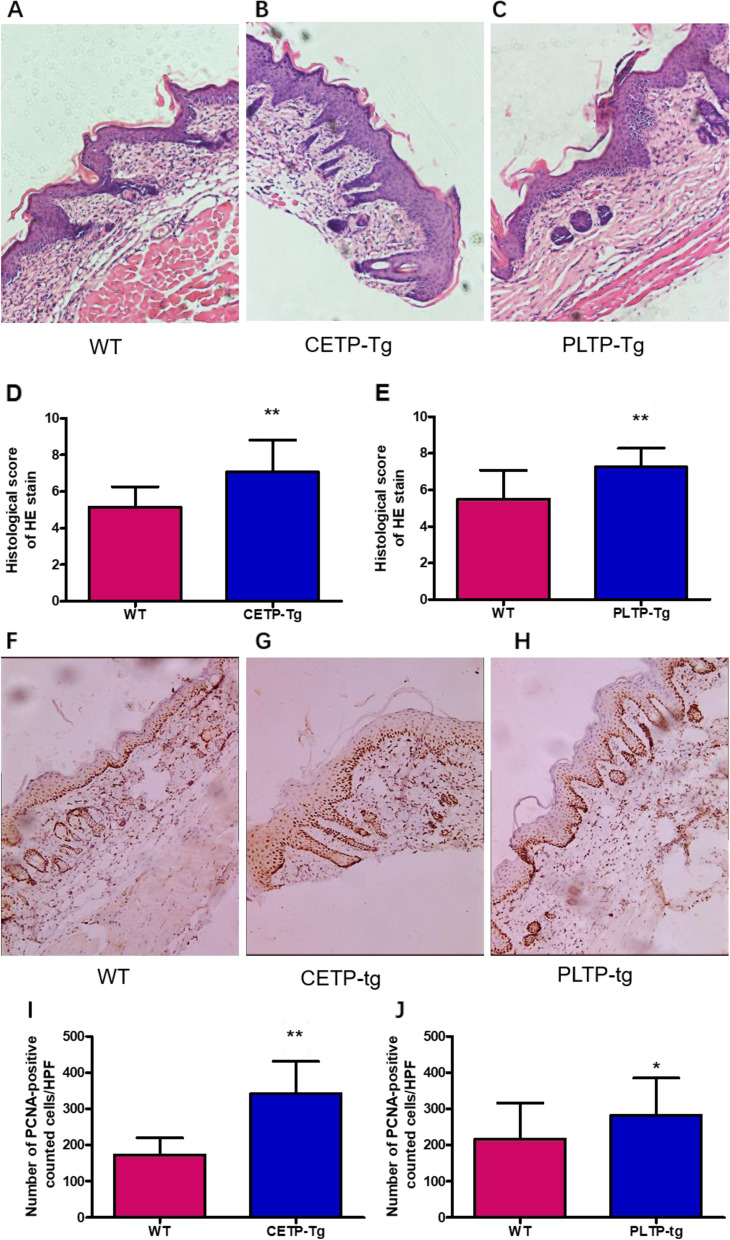
Imiquimod treatment alters epidermal thickening and differentiation and inflammatory factor infiltration. Back skin shaved in ETP-Tg, PLTP-Tg, and WT mice was exposed to IMQ cream. **A**–**C** HE staining of mouse back skin. **D**, **E** Histopathological score means ± standard deviations (SD). **F**–**H** PCNA protein immunohistochemical staining (back skin). **I**, **J** Calculation of PCNA-positive cell number within means±SDs from six typical high-power fields (HPF) from diverse mice. *N* = 6/group; * *P <* 0.05, ** *P <* 0.01 vs. WT group; IMQ, imiquimod; PCNA, CETP, cholesteryl ester transfer protein; PLTP, phospholipid transfer protein; WT, wild type; Tg, transgenic

HE-stained sections collected in IMQ-mediated skin revealed a significant difference in histopathological score between CETP-Tg, PLTP-Tg mice, and WT mice (Fig. 2A-C). Compared to WT mice, the histopathological score of CETP-Tg and PLTP-Tg mice increased considerably (Fig. 2 D, E). H&E sections exhibited epidermal thickening, and abundant infiltrates of mononuclear cells were observed. Figure 2 F-H depicts the expression of the PCNA protein, a widely used cell proliferation indicator, in the back samples. According to immunohistochemistry, cells that expressed PCNA were enriched into spinous and basal keratinocytes. IMQ challenge significantly increased PCNA protein expression in CETP-Tg and PLTP-Tg mice relative to their WT counterparts (Fig. 2I, J).

### Evaluation of IMQ-mediated skin inflammation

**Fig. 3:**
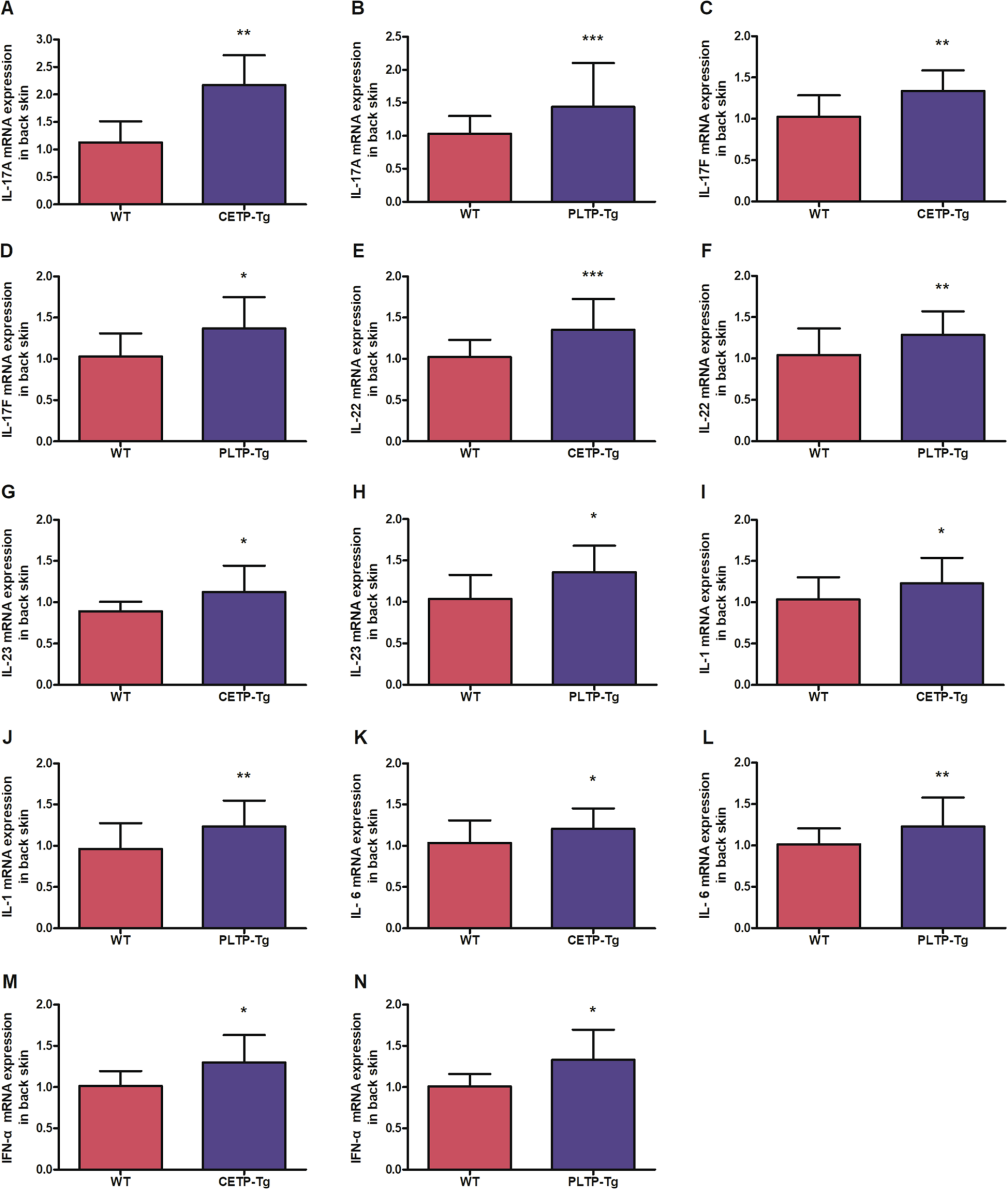
Imiquimod induces cytokine mRNA expression in the back skin. Back skin shaved in CETP-Tg, PLTP-Tg, and WT mice was exposed to IMQ cream treatment. After extracting RNA from back skin, (**A**, **B**) IL-17A, (**C**, **D**) IL-17F, (**E**, **F**) IL-22, (**G**, **H**) IL-23p19, (**I**, **J**) IL-1β, (**K**, **L**) IL-6, and (**M**, **N**) IFN-α expression was measured through qRT–PCR. GAPDH mRNA was used as the normalization reference. Symbols indicate the mean ± SD; *n* = 6/group; * *P* < 0.05, ** *P* < 0.01 vs. WT group; IMQ, imiquimod; IL, interleukin; PLTP, phospholipid transfer protein; CETP, cholesteryl ester transfer protein; WT, wild type; Tg, transgenic

Skin cytokine levels, including IFN-α, IL-1β, IL-6, IL-17A, IL-17F, IL-22, and IL-23p19, were detected to evaluate IMQ-mediated skin inflammation (Fig. 3A-N). We discovered that IMQ application increased IFN-α, IL-1β, IL-6, IL-17A, IL-17F, IL-22, and IL-23p19 mRNA levels in CETP-Tg and PLTP-Tg mice compared with WT mice.

### Evaluation of IMQ-mediated plasma inflammation

**Fig. 4:**
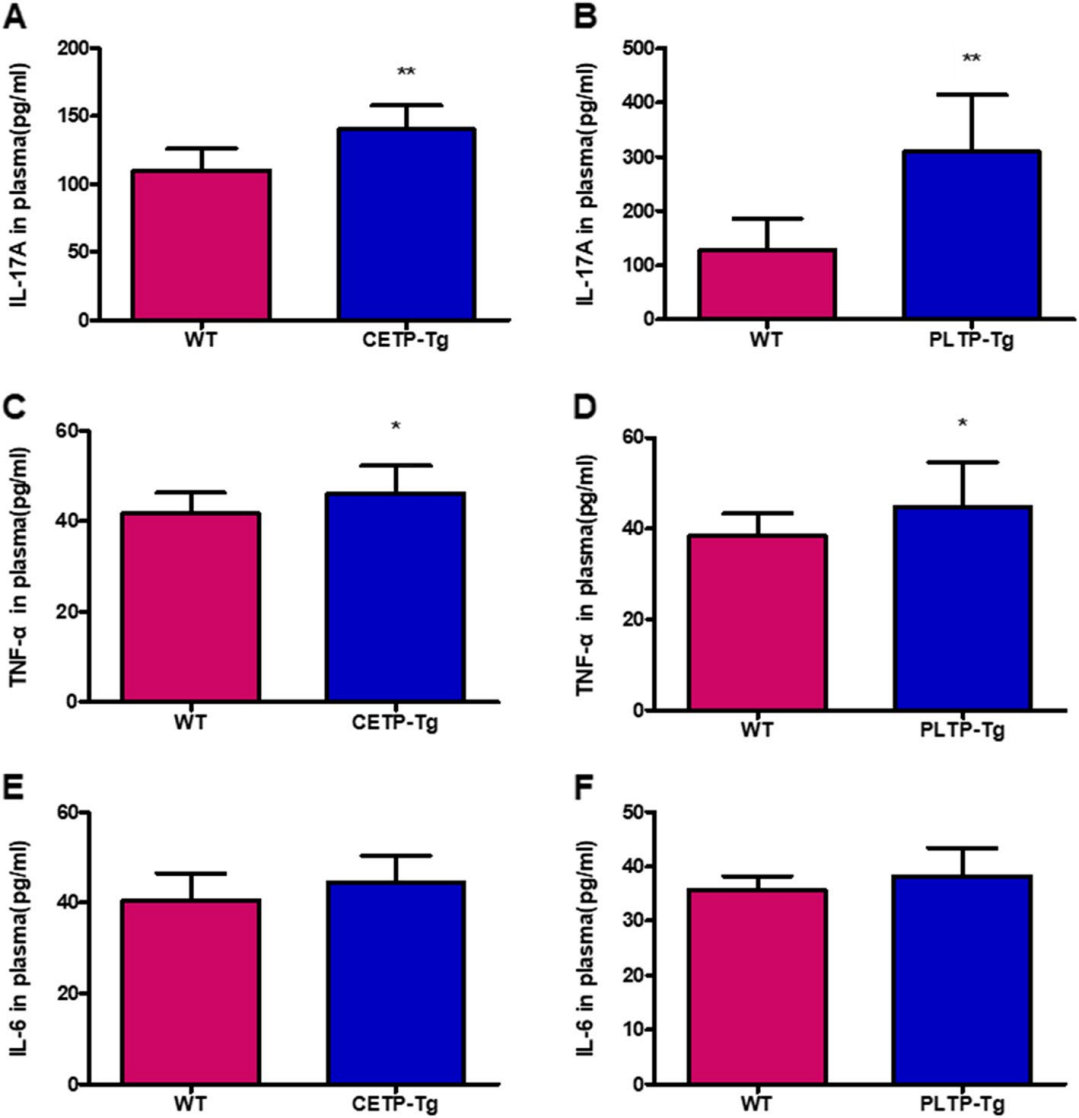
Imiquimod induces cytokine expression in the plasma. Back skin shaved in ETP-Tg, PLTP-Tg, and WT mice was subjected to IMQ cream treatment. Plasma expression of (**A**, **B**) IL-17A, (**C**, **D**) TNF-α, and (**E**, **F**) IL-6 was determined using ELISA kits. Symbols indicate the mean ± SD; *n* = 6/group; * *P* < 0.05, ** *P* < 0.01 vs. WT group; IMQ, imiquimod; IL, interleukin; PLTP, phospholipid transfer protein; CETP, cholesteryl ester transfer protein; WT, wild type; Tg, transgenic

In this study, the influences of CETP and PLTP on IMQ-mediated inflammation were determined. Figure 4A–F depicts plasma cytokine levels. IMQ challenge markedly elevated IL17A and TNF-α expression in the plasma of CETP-Tg and PLTP-Tg mice relative to WT mice (Fig. 4A–D). However, IL-6 was increased in CETP-Tg and PLTP-Tg mice compared with WT mice, although the difference was not statistically significant (Fig. 4E, F).

## Discussion

This study discovers that increased CETP and PLTP aggravates psoriasis in an imiquimod-induced mouse model. Its mechanism of action is possibly related to the inflammatory alterations and dyslipidemia induced by increased expression of CETP and PLTP in mice with psoriasis.

Psoriasis patients are typically dyslipidemic [26]. Recent studies have reported that psoriasis decreases cholesterol efflux capacity as well as HDL composition [6, 27, 28]. A previous study reported that HDL’s antioxidant and anti-inflammatory effects decreased in psoriasis. Both CETP and PLTP continuously modulate plasma HDL particles. CETP delivers CE to lipoproteins that contain apo B from HDL for remodeling HDL particles. Furthermore, during lipolysis, PLTP delivers phospholipids to HDL from triglyceride (TG)-abundant lipoproteins [29]. CETP-Tg and PLTP-Tg mice have significantly low HDL levels and a high risk of developing atherosclerosis. Both are the best models for studying the relationship between lower HDL and psoriasis, so CETP-Tg and PLTP-Tg mice with low HDL were selected as the subjects. CETP is regarded as a marker of liver side effects of acitretin treatment in psoriasis [30]. However, the CETP status in psoriasis may not be clear. Previous studies have shown that CETP is decreased in some patients with psoriasis and increased in others [31]. The present study found that increased CETP could aggravate psoriasis, suggesting that nonlipid transfer properties of CETP and confounders or modifiers may take part in that interplay. IMQ-mediated mouse skin is widely documented to mimic plaque-type psoriatic lesions in humans, accompanied by scaling, erythema, thickness, parakeratosis, acanthosis, neoangiogenesis, and inflammatory infiltrates that contain neutrophils, dendritic cells (DCs), plasmacytoid DCs (pDCs), and T cells. The immunological changes in the skin inflammation model induced by IMQ are highly clear [22]. Furthermore, excessive clinical topical IMQ treatment can result in psoriasis lesions, which are serious adverse reactions. Thus, the IMQ skin model was selected as the mouse model. Consistent with a previous report [32], IMQ-induced psoriatic lesions, such as scaling, erythema, acanthosis, thickening, neoangiogenesis, and inflammatory infiltrates, appeared in WT mice and CETP-Tg and PLTP-Tg mice. Furthermore, the present study discovered that CETP-Tg and PLTP-Tg mice have more severe pathological features with H&E staining than WT mice. Abnormally proliferating keratinocytes have been identified as a well-known feature of psoriasis. PCNA accounts for the credible factor for presenting cell growth [33]. Immunohistochemical analysis of PCNA localization within the epidermis showed that PCNA-positive cells accumulated and increased within spinous and basal keratinocytes in PLTP-Tg and CETP-Tg mice compared to WT mice.

Inflammation influences psoriasis lesions and the physiological status of the whole body. Numerous cytokines, including IFN-α, TNF-α, IL-1β, IL-6, IL-17A, IL-17F, IL-22, and IL-23p19, are overexpressed in local psoriatic skin or blood. IMQ also induces these cytokines similar to those involved in the pathogenesis of psoriasis. As a strong immune activator, IMQ serves as a TLR7/TLR8 ligand. IMQ-mediated psoriasis is IL-23/IL-17 axis-dependent [22]. IL-23, generated via keratinocytes and DCs within psoriatic lesions, enhances Th17-cell activation [34]. Then, Th17 cells are induced to secrete IL-17A and IL-17F, together with IL-22 [35], and the former two are responsible for neutrophil recruitment into tissues [36]. In addition, IL-22 promotes epidermal hyperplasia and dermal inflammation [37]. IL-6 can enhance neutrophil recruitment but also enhance keratinocyte proliferation [38, 39]. In the early stages of psoriasis, pDCs produce IFN-α [40]. Stressed keratinocytes produce IL-1β and TNF-α, which contribute to DC activation. Combining IL-6 and IL-1β drives naïve CD4+ cell differentiation to the Th17-cell subtype [41]. Overall, IMQ stimulates pDCs to produce IFN-α, IL-23, and TNF-α. Once activated, pDCs present an antigen that is not detected for naïve CD4+/CD8+ T cells while promoting cell differentiation to Th1 cells as well as IL-23-mediated Th17 cells. Following interaction with Th1 cells, pDCs and γδT cells act on keratinocytes, and IL-6, IL-17A, IL-17F, TNF-α, and IL-22 are secreted within the dermis. These cytokines result in hyperproliferation of keratinocytes and impede keratinocyte differentiation [42]. Both CETP and PLTP increase plasma cytokines [43, 44]. Hence, those cytokines were detected, and it was discovered that IMQ application significantly increased TNF-α, IFN-α, IL-1β, IL-6, IL-17A, IL-17F, and IL-22, together with the expression of IL-23p19 mRNA in the lesions among PLTP-Tg and CETP-Tg mice compared with WT mice [45]. Additionally, IL17A and TNF-α, but not IL-6, were found to be more abundant in the plasma of PLTP-Tg and CETP-Tg mice than in their WT counterparts. Additionally, IL-6 expression was elevated in CETP-Tg and PLTP-Tg mice. The above data are consistent with previous experimental results [46].

### Comparisons with other studies and what does the current work add to the existing knowledge

Previous documented studies have focused on the relationship between lipoprotein content and psoriasis, suggesting decreased HDL levels accompany psoriasis. Compared with other studies, the novelty of the present study is that both CETP and PLTP, the key proteins for lipid transfer and lipoprotein metabolism, affect pathological alterations in psoriasis.

### Study strengths and limitations

The overexpression of both CETP and PLTP aggravated the pathological and clinical scores. Furthermore, certain limitations should be noted in this work. Because of the small sample size, potentially important changes might be missed. It would be beneficial to enlarge the sample size to discover new findings. More than one psoriatic mouse model could be used to evaluate the relationship between lipid transfer protein expression and pathological changes in psoriasis in the future.

## Conclusion

The present study demonstrated that elevated expression of CETP and PLTP could aggravate the clinical and pathohistological characteristics and inflammation in mice with psoriasis. The relevant mechanisms could include decreased plasma levels and disordered function of HDL induced by overexpression of CETP and PLTP. Statins can alleviate clinical features in psoriatic patients. CETP inhibitors could increase HDL levels. Atorvastatin decreases plasma preβHDL by decreasing PLTP activity. The inhibition of CETP and PLTP could be expected to treat psoriasis with dyslipidemia in the future.

## Data Availability

Data and materials in this present manuscript are freely accessible.

## References

[1] Xie X, Zhang L, Li X, Liu W, Wang P, Lin Y, Han X, Li P (2021). Liangxue Jiedu formula improves psoriasis and dyslipidemia comorbidity via PI3K/Akt/mTOR pathway. Front Pharmacol.

[2] Miller IM, Ellervik C, Yazdanyar S, Jemec GB (2013). Meta-analysis of psoriasis, cardiovascular disease, and associated risk factors. J Am Acad Dermatol.

[3] Hao Y, Zhu YJ, Zou S, Zhou P, Hu YW, Zhao QX, Gu LN, Zhang HZ, Wang Z, Li J (2021). Metabolic syndrome and psoriasis: mechanisms and future directions. Front Immunol.

[4] Sachinidis A, Nikolic D, Rizzo M, Cianflone D (2019). Psoriasis and acute coronary syndrome risk. Int J Cardiol.

[5] Shih CM, Chen CC, Chu CK, Wang KH, Huang CY, Lee AW. The roles of lipoprotein in psoriasis. Int J Mol Sci. 2020;21(3):859. 10.3390/ijms21030859 PMC7036823 32013194

[6] Holzer M, Wolf P, Curcic S, Birner-Gruenberger R, Weger W, Inzinger M, El-Gamal D, Wadsack C, Heinemann A, Marsche G (2012). Psoriasis alters HDL composition and cholesterol efflux capacity. J Lipid Res.

[7] Holzer M, Wolf P, Inzinger M, Trieb M, Curcic S, Pasterk L, Weger W, Heinemann A, Marsche G (2014). Anti-psoriatic therapy recovers high-density lipoprotein composition and function. J Invest Dermatol.

[8] Salerno AG, Patrício PR, Berti JA, Oliveira HC (2009). Cholesteryl ester transfer protein (CETP) increases postprandial triglyceridaemia and delays triacylglycerol plasma clearance in transgenic mice. Biochem J.

[9] Harada LM, Amigo L, Cazita PM, Salerno AG, Rigotti AA, Quintão EC, Oliveira HC (2007). CETP expression enhances liver HDL-cholesteryl ester uptake but does not alter VLDL and biliary lipid secretion. Atherosclerosis.

[10] Deng S, Liu J, Niu C (2022). HDL and cholesterol Ester transfer protein (CETP). Adv Exp Med Biol.

[11] Borggreve SE, Hillege HL, Wolffenbuttel BH, de Jong PE, Zuurman MW, van der Steege G, van Tol A, Dullaart RP (2006). An increased coronary risk is paradoxically associated with common cholesteryl ester transfer protein gene variations that relate to higher high-density lipoprotein cholesterol: a population-based study. J Clin Endocrinol Metab.

[12] Marschang P, Sandhofer A, Ritsch A, Fiŝer I, Kvas E, Patsch JR (2006). Plasma cholesteryl ester transfer protein concentrations predict cardiovascular events in patients with coronary artery disease treated with pravastatin. J Intern Med.

[13] Marotti KR, Castle CK, Boyle TP, Lin AH, Murray RW, Melchior GW (1993). Severe atherosclerosis in transgenic mice expressing simian cholesteryl ester transfer protein. Nature.

[14] Plump AS, Masucci-Magoulas L, Bruce C, Bisgaier CL, Breslow JL, Tall AR (1999). Increased atherosclerosis in ApoE and LDL receptor gene knock-out mice as a result of human cholesteryl ester transfer protein transgene expression. Arterioscler Thromb Vasc Biol.

[15] Zhang M, Zhai X, Li J, Albers JJ, Vuletic S, Ren G (2018). Structural basis of the lipid transfer mechanism of phospholipid transfer protein (PLTP). Biochim Biophys Acta Mol Cell Biol Lipids.

[16] Ha YC, Barter PJ (1982). Differences in plasma cholesteryl ester transfer activity in sixteen vertebrate species. Comp Biochem Physiol B.

[17] Hayek T, Chajek-Shaul T, Walsh A, Agellon LB, Moulin P, Tall AR, Breslow JL (1992). An interaction between the human cholesteryl ester transfer protein (CETP) and apolipoprotein A-I genes in transgenic mice results in a profound CETP-mediated depression of high density lipoprotein cholesterol levels. J Clin Invest.

[18] Oliveira H, Raposo HF (2020). Cholesteryl Ester transfer protein and lipid metabolism and cardiovascular diseases. Adv Exp Med Biol.

[19] Jiang XC, Yu Y (2021). The role of phospholipid transfer protein in the development of atherosclerosis. Curr Atheroscler Rep.

[20] Zhang M, Zhai X, Li J, Albers JJ, Vuletic S, Ren G (2018). Structural basis of the lipid transfer mechanism of phospholipid transfer protein (PLTP). Biochim Biophys Acta Mol Cell Biol Lipids.

[21] van Haperen R, Samyn H, Moerland M, van Gent T, Peeters M, Grosveld F, van Tol A, de Crom R (2008). Elevated expression of phospholipid transfer protein in bone marrow derived cells causes atherosclerosis. PLoS One.

[22] Song C, Yang C, Meng S, Li M, Wang X, Zhu Y, Kong L, Lv W, Qiao H, Sun Y (2021). Deciphering the mechanism of Fang-Ji-Di-Huang-decoction in ameliorating psoriasis-like skin inflammation via the inhibition of IL-23/Th17 cell axis. J Ethnopharmacol.

[23] Gangadevi V, Thatikonda S, Pooladanda V, Devabattula G, Godugu C (2021). Selenium nanoparticles produce a beneficial effect in psoriasis by reducing epidermal hyperproliferation and inflammation. J Nanobiotechnology.

[24] Schaper K, Dickhaut J, Japtok L, Kietzmann M, Mischke R, Kleuser B, Bäumer W (2013). Sphingosine-1-phosphate exhibits anti-proliferative and anti-inflammatory effects in mouse models of psoriasis. J Dermatol Sci.

[25] Baker BS, Brent L, Valdimarsson H, Powles AV (1992). al-Imara L, Walker M, fry L: is epidermal cell proliferation in psoriatic skin grafts on nude mice driven by T-cell derived cytokines. Br J Dermatol.

[26] Nowowiejska J, Baran A, Flisiak I. Aberrations in lipid expression and metabolism in psoriasis. Int J Mol Sci. 2021;22(12):6561. 10.3390/ijms22126561 PMC8234564 34207318

[27] Tom WL, Playford MP, Admani S, Natarajan B, Joshi AA, Eichenfield LF, Mehta NN (2016). Characterization of lipoprotein composition and function in pediatric psoriasis reveals a more Atherogenic profile. J Invest Dermatol.

[28] Lee HJ, Han KD, Park HE, Han JH, Bang CH, Park YM, Lee JH (2021). Changes in metabolic syndrome and risk of psoriasis: a nationwide population-based study. Sci Rep.

[29] Yamashita S, Sakai N, Hirano K, Ishigami M, Maruyama T, Nakajima N, Matsuzawa Y (2001). Roles of plasma lipid transfer proteins in reverse cholesterol transport. Front Biosci.

[30] Nowowiejska J, Baran A, Krahel JA, Kamiński TW, Maciaszek M, Flisiak I. Serum cholesteryl Ester transfer protein (CETP) and Sortilin (SORT) in patients with psoriasis with relation to systemic treatment. Metabolites. 2022;12(4):340. 10.3390/metabo12040340 PMC9032539 35448527

[31] Torkhovskaia TI, Fortinskaia ES, Ivanova LI, Nikitina NA, Zakharova TS, Kochetova MM, ZhI K (2002). GIa S, Khalilov EM: [characteristics of the lipid transport system in psoriasis]. Voprosy meditsinskoi khimii.

[32] Van Belle AB, de Heusch M, Lemaire MM, Hendrickx E, Warnier G, Dunussi-Joannopoulos K, Fouser LA, Renauld JC, Dumoutier L (1950). IL-22 is required for imiquimod-induced psoriasiform skin inflammation in mice. J Immunol (Baltimore Md).

[33] Ma LJ, You Y, Bai BX, Li YZ (2009). Therapeutic effects of heme oxygenase-1 on psoriasiform skin lesions in guinea pigs. Arch Dermatol Res.

[34] Trakaki A, Marsche G. High-density lipoprotein (HDL) in allergy and skin diseases: focus on Immunomodulating functions. Biomedicines. 2020;8(12):558. 10.3390/biomedicines8120558 PMC7760586 33271807

[35] Friedrich M, Krammig S, Henze M, Döcke WD, Sterry W, Asadullah K (2000). Flow cytometric characterization of lesional T cells in psoriasis: intracellular cytokine and surface antigen expression indicates an activated, memory/effector type 1 immunophenotype. Arch Dermatol Res.

[36] Schlaak JF, Buslau M, Jochum W, Hermann E, Girndt M, Gallati H (1994). Meyer zum Büschenfelde KH, Fleischer B: T cells involved in psoriasis vulgaris belong to the Th1 subset. J Invest Dermatol.

[37] Boniface K, Blom B, Liu YJ, de Waal MR (2008). From interleukin-23 to T-helper 17 cells: human T-helper cell differentiation revisited. Immunol Rev.

[38] Romani L, Mencacci A, Cenci E, Spaccapelo R, Toniatti C, Puccetti P, Bistoni F, Poli V (1996). Impaired neutrophil response and CD4+ T helper cell 1 development in interleukin 6-deficient mice infected with Candida albicans. J Exp Med.

[39] Hurst SM, Wilkinson TS, McLoughlin RM, Jones S, Horiuchi S, Yamamoto N, Rose-John S, Fuller GM, Topley N, Jones SA (2001). Il-6 and its soluble receptor orchestrate a temporal switch in the pattern of leukocyte recruitment seen during acute inflammation. Immunity.

[40] Lande R, Gregorio J, Facchinetti V, Chatterjee B, Wang YH, Homey B, Cao W, Wang YH, Su B, Nestle FO (2007). Plasmacytoid dendritic cells sense self-DNA coupled with antimicrobial peptide. Nature.

[41] Elloso MM, Gomez-Angelats M, Fourie AM (2012). Targeting the Th17 pathway in psoriasis. J Leukoc Biol.

[42] Wagner EF, Schonthaler HB, Guinea-Viniegra J, Tschachler E (2010). Psoriasis: what we have learned from mouse models. Nat Rev Rheumatol.

[43] Shelly L, Royer L, Sand T, Jensen H, Luo Y (2008). Phospholipid transfer protein deficiency ameliorates diet-induced hypercholesterolemia and inflammation in mice. J Lipid Res.

[44] Dusuel A, Deckert V, Pais de Barros JP, van Dongen K, Choubley H, Charron É, Le Guern N, Labbé J, Mandard S, Grober J (2021). Human cholesteryl ester transfer protein lacks lipopolysaccharide transfer activity, but worsens inflammation and sepsis outcomes in mice. J Lipid Res.

[45] Grän F, Kerstan A, Serfling E, Goebeler M, Muhammad K (2020). Current developments in the immunology of psoriasis. Yale J Biol Med.

[46] Tokuyama M, Mabuchi T. New treatment addressing the pathogenesis of psoriasis. Int J Mol Sci. 2020;21(20):7488. 10.3390/ijms21207488 PMC7589905 33050592

